# How a 7-Week Food Literacy Cooking Program Affects Cooking Confidence and Mental Health: Findings of a Quasi-Experimental Controlled Intervention Trial

**DOI:** 10.3389/fnut.2022.802940

**Published:** 2022-03-17

**Authors:** Joanna Rees, Shih Ching Fu, Johnny Lo, Ros Sambell, Joshua R. Lewis, Claus T. Christophersen, Matthew F. Byrne, Robert U. Newton, Siobhan Boyle, Amanda Devine

**Affiliations:** ^1^School of Medical and Health Sciences, Institute for Nutrition Research, Edith Cowan University, Joondalup, WA, Australia; ^2^School of Medical and Health Sciences, Edith Cowan University, Joondalup, WA, Australia; ^3^School of Science, Edith Cowan University, Joondalup, WA, Australia; ^4^School of Electrical Engineering, Computing and Mathematical Sciences, Curtin University, Perth, WA, Australia; ^5^Medical School, University of Western Australia, Perth, WA, Australia; ^6^School of Public Health, University of Sydney, Sydney, NSW, Australia; ^7^Western Australian Human Microbiome Collaboration Centre, School of Molecular and Life Sciences, Curtin University, Perth, WA, Australia; ^8^Centre for Integrative Metabolomics and Computational Biology, Edith Cowan University, Perth, WA, Australia; ^9^School of Education, Edith Cowan University, Joondalup, WA, Australia; ^10^School of Medical and Health Sciences, Exercise Medicine Research Institute, Edith Cowan University, Joondalup, WA, Australia; ^11^School of Human Movement and Nutrition Sciences, University of Queensland, Brisbane, QLD, Australia; ^12^The Good Foundation, Melbourne, VIC, Australia

**Keywords:** 7-week cooking program, food literacy learning, self-esteem (SE), mental health related quality of life, cooking confidence, dietary intake and consumption pattern

## Abstract

Obesity and mental health disorders are rising simultaneously with shifting dietary behavior away from home cooking, toward typically nutrition-poor and energy-dense convenience meals. Food literacy strongly influences nutrition choices. Community-based cooking interventions target barriers to healthy eating and facilitate development of food literacy skills, thereby potentially increasing preparation of home-cooked meals and positively influencing health. This study of 657 healthy Australian adults explored the efficacy of a 7-week cooking program in improving cooking confidence, whether this transferred to behavior surrounding food, and/or affected mental health. Significant post-program improvements in cooking confidence and satisfaction (all *p* < 0.001, $\eta_{p}^{2}$ 1.12 large), ability to change eating habits (*p* < 0.001) and overcome lifestyle barriers (*p* = 0.005) were observed for the intervention group but not control. Participation also improved mental and general health (all *p* < 0.05, $\eta_{p}^{2}$ 0.02 small). No changes were observed for acquisition and consumption of food, or nutrition knowledge in either group. This 7-week cooking program built cooking confidence and improved general and mental health but did not change dietary behavior. To further improve nutrition related behaviors associated with better mental health, more effort is needed to recruit those with below-average nutrition knowledge and interest in cooking.

## Background

Over the past few decades Australia has experienced fundamental changes to the behaviors associated with food acquisition, preparation and consumption (1, 2). Numerous studies have demonstrated that the changes to the built and food environment have impacted physical activity, healthful eating and obesity (3). For the majority of the population the living environment features significant barriers to a healthy lifestyle (2, 4–6). These include the community, consumer, worksite/school and home food environments that discourage healthy dietary patterns, typically stocking a greater proportion of energy dense, nutrient poor foods (7). This has led to the loss of skills needed for simple, healthy home cooking (3, 8–10). Increasing pressures of busy work-life schedules have created a demand for convenience and fast foods and a parallel decline in the consumption of fresh, home-cooked, healthy meals (10–13). In response, there has been substantial growth within the food industry of ready-made meals, often energy-dense, nutrient-poor, with high levels of salt, saturated fat and sugar (6, 11, 14, 15). These are typical foods that comprise a “Western Diet” (14, 16–18), which has been associated with declining physical (4, 19) and mental health (17, 19–24) at a population level, both globally and in Australia (16, 25).

Data from the (Australian) National Health Survey (NHS) 2017–18, reflect the “obesogenic” food environment as the number of Australian adults who were overweight or obese had risen by 3.6% over 3 years to 67.0% (12.5 million people) (26). Statistics suggest that the population is not adhering to the Australian Dietary Guidelines (27). In 2017–18, only 1 in 20 Australian adults (18 + yrs) met the guidelines for recommended daily serves of fruit and vegetables and discretionary food intake contributed to around one third of total energy intake (26) at the expense of more nutritious foods from the 5 food groups (2, 16). These trends have remained fairly consistent over time (25). The increasing uptake of highly processed Western diet patterns over fresh, home-cooked meals with high dietary fiber and nutrients, have been associated with higher rates of both metabolic disease and mental health disorders (20, 22, 28–33). This is of significance when NHS figures state that 1 in 5 adults had a mental health or behavioral condition during the 12 months prior to the survey (25). The Australian National Mental Health Commission propose considerable potential economic and social benefits could be gained from investment in promotion, prevention and early intervention for mental health reform (34). The economic cost of overweight and obesity is estimated at $11.8 billion, of which $5.4 billion is due to direct health costs such as disability and hospitalization (35).

Maintenance of a healthy diet, such as adherence to national dietary guidelines is central to lowering rates of overweight and obesity (36) and to reducing the risks of developing mental health disorders (21, 37, 38), cardiovascular disease (39–41), cancer (42) and other chronic diseases that are detrimental to health and wellbeing (32, 43). Food literacy underpins nutrition choices and is described as four domains of inter-related knowledge, skills and behaviors (plan and manage; select; prepare; and eat), which scaffold the ability of individuals and communities to maintain consistent diet quality and determine intakes (44). Higher self-perceived food literacy has been linked to better nutrition quality and is recognized as an important determinant of healthy eating (45). In addition, a 2018 survey conducted by Dietitians of Canada in a primary care setting, highlighted the potential for food literacy nutrition education for improving mental health in their patients (46). Various methods for measuring food literacy with respect to dietary intake have been proposed (45, 47), but discrepancies exist due to a lack of validated tools and consistency between studies (45, 48, 49). Factors such as age, gender, socioeconomic and cultural differences also need to be considered. More recently the context of cooking confidence has been separated into cooking skills confidence and food skills confidence (50).

In Australia, as elsewhere around the world, the investment in policy development to tackle the obesity epidemic and create a healthier food environment is gaining momentum (31, 35, 51). Promotion of positive nutrition messages to increase knowledge, change beliefs, motivation, skills and behaviors toward healthier dietary patterns and beneficial health outcomes are a priority (51–53). It has been reported that those with higher confidence in cooking are more likely to have healthier food behaviors, include more vegetables and have a body mass index (BMI) that is classified as being in the healthy weight category (14, 54, 55). Whereas, lower cooking confidence presents barriers to healthy eating such as the imposing influence of time constraints and greater tendency to use convenience foods (56, 57).

Community-based cooking programs create a knowledge translation and exchange platform that can provide a motivating and socially connected setting in which to build and benchmark knowledge, skills and best practice on achievable ways to have a healthier diet (48, 51, 55). A review of 22 Australian cooking skills interventions conducted over the past 20 years found that although most reported improved cooking confidence, evidence that participation improved dietary behavior was less conclusive (53). In spite of this, lower food literacy has been associated with lower self-rated cooking skills, negative attitudes surrounding the cost of healthy foods, lower fruit and vegetable intake and higher discretionary food consumption (58).

Apart from the convenience factor, two additional barriers to healthy eating are a lack of “knowing how to cook” (8) and the self-perception of increased cost and availability of fresh produce (58). Lack of cooking skills typically accompany limited cooking confidence and lower levels of satisfaction and cooking enjoyment (53, 59–61). Whereas, higher cooking confidence has been linked with greater enjoyment of the food and eating experience (8). This translates into food acquisition behaviors such as prioritizing healthier fresh foods over convenient, pre-processed alternatives in the weekly grocery shop (8, 61, 62). The perception that healthy foods are expensive and/or less readily available, presents an additional barrier to healthy eating that negatively influences food purchasing decisions and dietary intake (12). This is further amplified in rural and remote areas and those of lower socio-economic status and household income (55, 57, 63). Population nutrition literature states that low income or other economic constraints are a distinct issue in dietary behavior, as are a lack of food skills, however the two should not be conflated (53, 58). Begley et al. (58) suggest that it is critical for cooking skills interventions to recruit participants with low self-rated cooking skills, who consider healthy foods expensive and have poor dietary intakes. The challenge is therefore, to deliver community education programs with the capacity to reach these specific audiences. In recent times there has been a proliferation of community-based cooking interventions, aimed at targeting the barriers to healthy eating and incorporating the food literacy skills; plan and manage; and select (8, 13, 48, 64–66). Recent reviews of cooking skills interventions (8, 13) found that program duration varied greatly, as did outcome measures, with no clear gold standard emerging for improving dietary intake. In Australia, programs have tended to target specific population groups, such as vulnerable or indigenous groups or those with low income but who may not have low cooking confidence or food literacy (53, 64, 67). Therefore, to capture the desired target group, it may be necessary to recruit from a broader section of the general population. In addition, there have been limited studies that have explored the relationship between food literacy, mental health and other physiological biomarkers.

The Jamie's Ministry of Food (JMOF) Australia was a community-based program that commenced in Western Australia (WA) in 2016 and has been described in detail elsewhere (68). In brief, the JMOF program taught basic cooking and budgeting skills, efficient food shopping strategies, and skills to prepare healthy meals at low cost. The aim of the JMOF program was to help people prepare simple, fresh, healthy food quickly and cheaply. In WA, The Good Foundation (TGF), supported by Edith Cowan University (ECU), delivered the program over the 3-year partnership via a mobile kitchen, thereby extending the reach to a variety of areas of socioeconomic status. Researchers at ECU in association with TGF, designed a longitudinal study to examine the effects of the program.

The purpose of the study was to explore the impact of this community-based cooking program and whether it was efficacious in improving cooking confidence and ability that may then be transferred to behavior and attitudes surrounding food, including acquisition, preparation and consumption. The broader study included collection of food frequency questionnaire data for dietary intake, lifestyle and biomarker measures to further explore potential health benefits (68), in this paper we report on the preliminary evaluation of the broader study. The primary aim of the study was to determine whether the JMOF WA program could instigate change in participants' self-perceived cooking confidence. The secondary aim was to determine if the program instigated change in participants' nutrition related knowledge, attitudes, beliefs and behaviors toward healthy cooking and eating and associated general health and mental health outcomes. These aims were based on the premise that preventive nutrition, as delivered by community-based programs such as this one, along with healthy connections to others, would positively impact nutrition-related lifestyle behaviors, quality of life and health outcomes that could be sustained over time. The novel inclusion of mental health assessment will help to provide valuable insight into the potential relationship between cooking confidence, dietary intake and mental health. This also and responds to the Australian Government's National Obesity Prevention Strategy 2022–2032 [Draft] that acknowledges the strong link between poor dietary habits and poor mental health and includes a call for preventive strategies to target consumer options (Strategy 2.1) (69).

## Methods

### Participants

**Figure 1:**
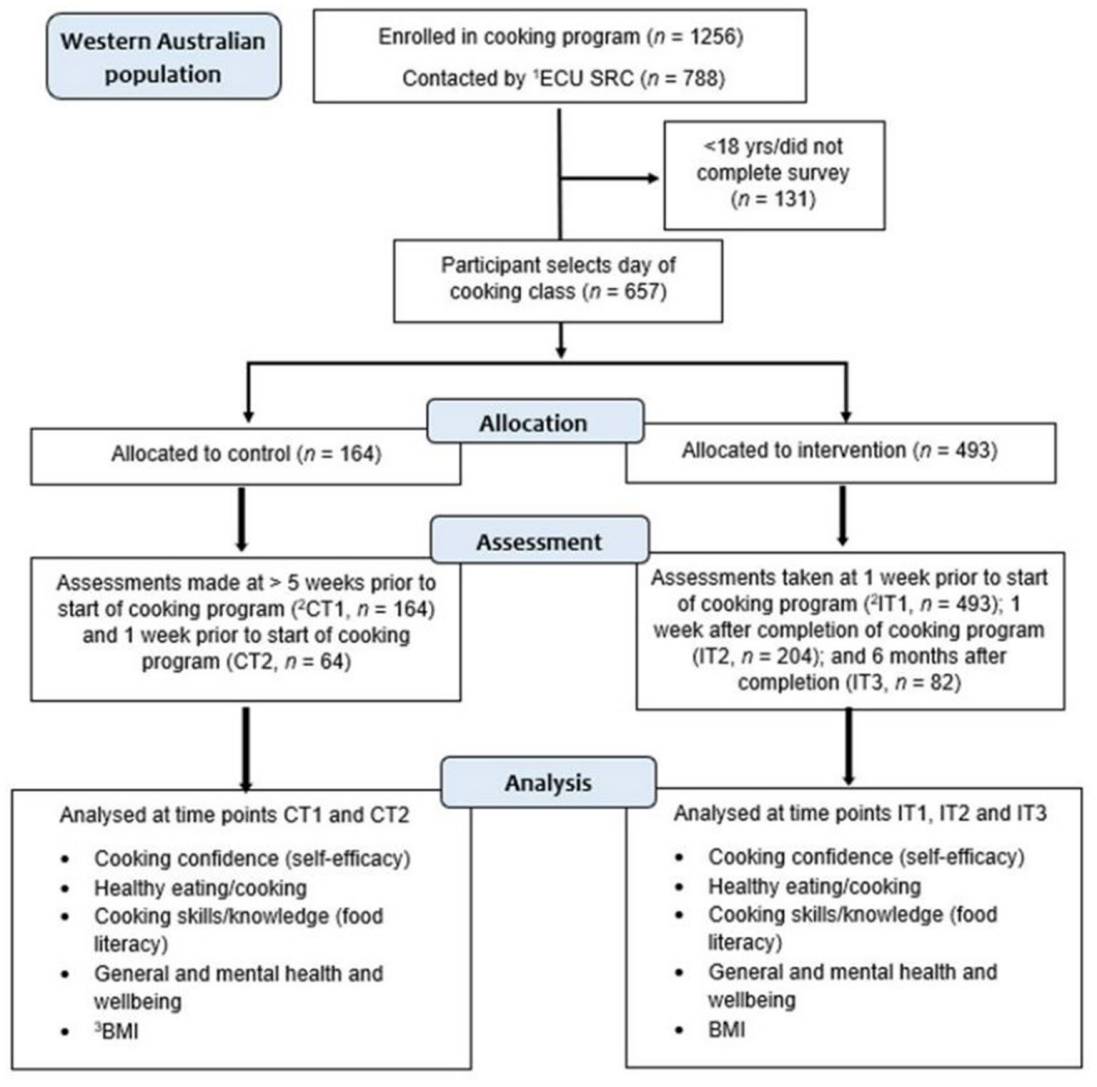
Study flow diagram of participant enrolment, allocation, assessment and analysis over time. ^1^ECU SRC, ECU Survey Research Centre; ^2^CT1, CT2, IT1, IT2, IT3, control timepoint 1 and 2, intervention timepoint 1, 2 and 3; ^3^BMI, body mass index.

This quasi-experimental controlled study involved the selection of an intervention and a control group from JMOF courses that were run in a total of 16 locations throughout WA. These locations included 11 metropolitan and 5 regional areas, selected for their lower socioeconomic status (Additional File 1, Part a). The intervention group participants from each location completed a cooking program consisting of weekly 90-min sessions for 7 weeks. The corresponding control group from each location were participants who were on the program waitlist and had yet to complete the program (Figure 1), known as a “waitlist control” (70, 71). The total study population was 657 of whom, 493 were intervention and 164 were control participants.

All program registrants aged >18 years were eligible to participate, and no other inclusion or exclusion criteria were applied. Registrant information for each course was provided by TGF to the ECU Survey Research Centre (ECU SRC) who contacted adult participants, determined those who were willing to participate and forwarded a link to the online consent form and the LAB survey. Withdrawal was an option at any stage of participation. Ethics approval was provided by the ECU Human Research Ethics Committee (HREC) (ID 15362: Newton) and all participants gave their consent, either written or online, prior to their involvement in the study.

A non-random sampling approach was adopted due to the philosophy of the JMOF program, which emphasized the importance of participants being able to attend when and with whom they preferred. Allocation to either the control or intervention group was based on the day of the week of their chosen session (Monday–Saturday). Participants who opted for a class on a Monday or Thursday were allocated to the control group. Remaining participants were allocated to the intervention group. The intervention involved participation in weekly cooking sessions over 7 weeks, delivered by a nutrition professional from the fully equipped JMOF mobile kitchen. The 90-min hands-on cooking session used a new recipe each week to scaffold learning of skills and knowledge about nutritious foods, to enable increased cooking confidence, skill development and food literacy knowledge. In addition, the participants were taught knife handling skills and were given tips and advice about healthy options for how to boost flavor and create dishes using fresh foods to replace pre-prepared ingredients. Food-budget planning, kitchen economy and reduction of food waste were also covered. The intervention group were surveyed at three time points and the control group were surveyed at two time points (Supplementary Material Additional File 1). As each course was promoted to the community 2 months before the program start date this left only an 8-week window in which to recruit participants to the cooking program and the ECULABJMOF study. As the control group comprised registrants who were waiting to start their program, it was only feasible to survey the control group twice, over a 5-week period, during the time leading up to program commencement (Supplementary Material Additional File 1). Further details on the study protocol can be accessed in previous literature (68, 72, 73). Sample size calculations were based on previous studies of the JMOF program (62).

### Data Collection

Data was collected between September 2016 and June 2019 using the “standard” JMOF participant questionnaire (from this point forward referred to as LAB survey), an online self-report questionnaire used in previous JMOF evaluations (62, 72, 74). Our study differs from earlier evaluations by the inclusion additional sections described below, to explore mental health and wellbeing (68). Briefly, the questionnaire gathered demographic information including BMI (calculated from self-reported height and weight) and included 6 questions on habitual dietary patterns and behavior; 2 multi-item 5-point Likert scale questions on cooking confidence and cooking satisfaction based on cooking skills; 3 questions on nutrition knowledge; and 6 questions on household food spending and eating behaviors (72). In addition, it incorporated the validated Rosenburg Global Self Esteem scale (RGSE) (75, 76) and a 5-point Likert scale question on self-reported general health (excellent to poor). Our study included further assessments of mental health, subjective vitality and wellbeing that included the SF-12 Health Survey (77), the Subjective Vitality Scale (SVS) (78, 79) and the Warwick Edinburgh Mental wellbeing Scale (WEMBWS) (80, 81). Details of the questionnaire, the logic model on which it was based, and the validation of the tools included have been described in detail in an earlier publication (68, 72, 73).

The primary outcome was cooking confidence (operationalised as personal beliefs of self-efficacy), explored in terms of change over time and between the intervention and control study groups. The five areas measured were:

1. Confidence About Being Able to Cook From Basic Ingredients.
2. Confidence About Following a Simple Recipe.
3. Confidence About Preparing and Cooking new Foods and Recipes.
4. Confidence That What Is Cooked Will “Turn out Well.”
5. Confidence About Tasting Foods not Eaten Before.

Scores from the five components were summed together to give an overall confidence score. Secondary outcomes were similarly explored within and between groups and over time. The secondary outcomes were selected to address attitudes and beliefs regarding food acquisition, preparation and consumption. These included healthy cooking; healthy eating; affordability of a healthy meal; social connectedness around cooking and eating; healthy eating habits; cooking ability, enjoyment and satisfaction; and self-reported nutrition knowledge. Additional secondary outcomes included self-perceived general and mental health and wellbeing; and self-reported BMI.

Flego et al. (62) reported a significant increase in the intervention group across all of the confidence measures (all *p* < 0.001) with a minimum increase of 0.53 on a 5-point scale, whilst the control group saw minimal changes (all *p* = 0.22). Further, the minimum difference in change between the control and intervention group corresponded to a medium Cohen's effect size (*d* = 0.46). The difference in change in the *overall* confidence (5–25 scales) between the groups was 3.31 (*p* < 0.001), with an estimated Cohen's effect of 0.9 (i.e., a large effect size). Given this, an *a-priori* sample size was calculated for a mixed model repeated-measures design (2 groups and 3 time points) to detect at least a medium interaction effect size (Cohen's *f*^2^ = 0.25) at 1% level of significance (adjusting for multiple outcomes) and 80% power using G^*^Power. The minimum required sample size was 40 (or 20 per group). After accounting for 20% attrition rate, the adjusted minimum sample size was 50 (or 25 per group).

### Statistical Methods

Statistical analysis was performed using IBM SPSS Statistics for Windows, version 25.0 (82). Descriptive statistics of normally distributed continuous variables were expressed as mean ± SD and non-normally distributed continuous variables as median, IQR. Categorical variables were expressed as number and proportion (%). The primary outcome of cooking confidence was explored in terms of change over time and between the intervention and control study groups. To test for key between-group differences at baseline, Chi-square and independent *t*-tests were applied as appropriate. General linear modeling (GLM) was used to test the presence and magnitude of any program intervention effect within and between groups over time (baseline T1 and 5/7-week follow-up T2). Adjusted analyses were conducted using the following covariates selected due to their significant relationship with the outcome variables; age, gender, BMI, household income per year before tax and highest level of education attained. T1 values were used for all covariates as the intervention period of 7 weeks (or control period of 5 weeks) was deemed unlikely to contribute a significant difference in individual circumstances that would cause any resultant influence on evaluation outcomes. To determine whether the intervention effect (if any) was maintained at 6 months (T3) for the intervention group, GLM for repeated measures was used as this method allowed for randomly missing follow-up data.

The secondary outcomes addressing attitudes and beliefs regarding food acquisition, preparation and consumption were analyzed using GLM and the same covariates used for the adjusted model. Attitudes and beliefs were measured using a 4-item Likert scale ranging from “strongly disagree” to “strongly agree” (Supplementary Material Additional File 2). To explore social connectedness in greater detail, we included an additional adjustment for whether the participant attended with others or on their own. A stratified analysis was conducted to examine differences in the program's effect on cooking confidence and mental health outcomes (General health, GSE, MCS, SVS and WEMWBS) between sexes, income levels and education status. Self-reported nutrition knowledge about salt, fat and sugar was examined between the control and intervention groups, over time and within the intervention group, using a McNemar test. Results for all statistical analyses are reported as mean and change in mean scores by treatment (control vs. intervention) (with standard SD). Significance was achieved at *p* < 0.05 and effect size was reported as partial eta squared ($\eta_{p}^{2}$), where 0.01, 0.06, and 0.14 correspond to a small, medium and large effect size, respectively (83).

## Results

### Study Population Characteristics

The combined total of participant numbers for T1, T2, and T3, collected from each course are shown in Figure 1. Overall, at T1 there were 493 in the intervention group and 164 in the control group. At T2, retention in the intervention group was 41.4% (*n* = 204) and 39.0% in the control group (*n* = 64). There were 82 participants at T3 (intervention group only). No significant differences were observed between the control and intervention groups at T1 or at T2 (Supplementary Material Additional File 3) for any of the variables of interest. Participant characteristics by group are presented in Table 1. At T1, there was no difference in gender distribution between the control group (male 23.8% and female 76.2%) and intervention group [male 22.5% and female 77.5%; $\chi_{\left(1\right)}^{2}$ = 0.11, *p* = 0.738], nor age [44.4 ± 14.9 years vs. 43.9 ± 14.7 years; *t*_(651)_ = 0.388, *p* = 0.698]. Similarly, BMI (calculated from self-reported height and weight) was not different between the control and intervention groups [27.1 ± 6.2 vs. 27.5 ± 6.1 kg/m^2^, *t*_(595)_ = 0.71, *p* = 0.480]. Almost one third of participants had a BMI classification of overweight (32.5%) and nearly two thirds (59.5%) of all participants were overweight or obese. Almost two-thirds of participants in the control group (62.2%) and two-thirds in the intervention group (65.5%) attended with others, or as part of an organization or group. Significantly more participants in the intervention group (13.0%) attended as part of a community group than in the control group (2.0%) [$\chi_{\left(4\right)}^{2}$ = 14.8, *p* = 0.005] (Table 1).

**Table 1:** Detailed demographic characteristics of all participants by group and at each time point.

	**T1**		**T2**		**T3**
	**Control** **baseline (6 weeks pre-program)** ***n* = 164**	**Intervention** **baseline (1 week pre-program)** ***n* = 493**	**Control** **5-week follow-up (1 week pre-program)** ***n* = 64**	**Intervention** **7-week follow-up(immediately post-program)** ***n* = 208**	**Intervention** **(6 months post-program)** ***n* = 82**
**Sex**					
Female	125 (76.2)	382 (77.5)	50 (78.1)	154 (75.5)	58 (70.7)
Male	39 (23.8)	111 (22.5)	14 (21.9)	50 (24.5)	24 (29.3)
Age in years	44.4 ± 14.9	43.8 ±14.7	48.0 ±14.1	45.3 ±14.9	45.9 ±13.9
**Age group**					
18–24	10 (8.4)	48 (12.9)	2 (1.7)	16 (4.3)	6 (7.3)
25–34	23 (19.3)	68 (18.3)	4 (3.4)	32 (8.6)	12 (14.6)
35–44	25 (21.0)	85 (22.9)	8 (16.7)	28 (7.5)	20 (24.4)
45–54	32 (26.9)	83 (22.4)	14 (11.8)	34 (9.2)	21 (25.6)
55–64	14 (11.8)	58 (15.6)	6 (5.0)	28 (7.5)	17 (20.7)
65–74	11 (9.2)	24 (6.5)	6 (5.0)	14 (3.8)	5 (6.1)
75+	3 (2.5)	3 (0.8)	–	–	1 (1.2)
BMI kg/m2	27.1 ± 6.2	27.1 ± 4.2	27.5 ± 6.1	27.1 ± 6.8	26.4 ± 5.4
**Highest level of education completed**					
High school, year 12 or less	39 (32.8)	129 (34.8)	11 (9.2)	38 (10.2)	18 (22.0)
TAFE, apprenticeship, technical diploma or certificate	35 (29.4)	93 (25.1)	10 (8.4)	47 (12.7)	16 (19.5)
Tertiary, bachelor's degree or higher	45 (37.8)	149 (40.2)	19 (16.0)	67 (18.1)	48 (58.5)
**Current employment**					
Full time	38 (31.9)	119 (32.1)	15 (2.6)	51 (13.7)	28 (34.1)
Part time/casual	33 (27.7)	99 (26.7)	8 (6.7)	38 (10.2)	21 (25.6)
Retired	16 (13.4)	38 (10.2)	8 (6.7)	21 (5.7)	12 (14.6)
Home duties/carer	8 (6.7)	38 (10.2)	2 (1.7)	10 (2.7)	7 (8.5)
Not working (permanently ill/unable to work, unemployed)	4 (3.4)	28 (7.6)	2 (1.7)	10 (2.7)	2 (2.4)
Student (full time, part time)	14 (11.8)	33 (8.9)	3 (2.5)	16 (4.3)	7 (8.5)
Other	6 (5.0)	16 (4.3)	2 (1.7)	6 (1.6)	1 (1.2)
**Household yearly income**					
Nil	4 (3.4)	6 (1.6)	0 (0.0)	1 (0.7)	1 (1.2)
$1–$6,000	1 (0.8)	1 (0.3)	0 (0.0)	1 (0.7)	0 (0.0)
$6,001–$13,000	2 (1.7)	4 (1.1)	0 (0.0)	3 (2.0)	2 (2.4)
$13,001–$20,000	2 (1.7)	18 (4.9)	0 (0.0)	7 (4.6)	2 (2.4)
$20,001–$30,000	12 (10.1)	25 (6.7)	2 (5.0)	10 (6.6)	4 (4.9)
$30,001–$50,000	12 (10.1)	45 (12.1)	6 (15.0)	14 (9.2)	10 (12.2)
$50,001–$100,000	31 (26.1)	92 (24.8)	12 (30.0)	36 (23.7)	20 (24.4)
$100,001–$150,000	22 (18.5)	74 (19.9)	7 (17.5)	28 (18.4)	20 (24.4)
More than $150,000	19 (16.0)	57 (15.4)	8 (20.0)	34 (22.4)	15 (18.3)
Don't know	14 (11.8)	49 (13.2)	5 (12.5)	18 (11.8)	8 (9.8)
Household size, mean number of people	3.2 ± 1.8	3.3 ± 1.9	3.0 ± 1.7	3.06 ± 1.6	2.9 ± 1.3
**Who they attended with**					
Attended with others or as part of organization/group	102 (62.2)	323 (65.5)	31 (48.4)	137 (67.2)	49 (59.8)
Attended with a friend	33 (32.4)	94 (29.1)	10 (32.3)	40 (29.2)	14 (28.6)
Attended with family	61 (59.8)	151 (46.7)	17 (54.8)	65 (47.4)	26 (53.1)
Attended with a carer	4 (3.9)	30 (9.3)	0 (0.0)	8 (5.8)	4 (8.2)
Attended with a community group	2 (2.0)	42 (13.0)	0 (0.0)	9 (6.6)	2 (4.1)
Attended with other	2 (2.0)	6 (1.9)	4 (12.9)	15 (10.9)	3 (6.1)

*Continuous variables expressed as mean ± SD, categorical variables expressed as number and proportion (n %). TAFE, Technical and further education*.

### Cooking Confidence

For the intervention group, each of the individual components and the overall score for cooking confidence significantly improved over time whilst no change was observed in the control group, excepting cooking confidence in following a recipe. In this instance, an improvement was observed in the control group as well, however, it was not to the level of improvement observed in the intervention group (Table 2). Furthermore, for all five confidence questions, the improvements observed in the intervention group at T2 were maintained at T3 (Supplementary Material Additional File 4). In stratified analyses a significant difference between confidence gains T1 to T2 were observed between sexes (*p* = 0.021) but not between levels of education (*p* = 0.143) nor income (*p* = 0.466). At T1, female participants were more confident about cooking than males (*p* < 0.01), but at T2 both genders were equally confident about their cooking skills and ability to follow a simple recipe (*p* = 0.39) (Supplementary Material Additional File 4).

**Table 2:** Multivariable-adjusted general linear models of program interaction effects over time on cooking confidence components at T1 (baseline), T2 (5/7-week follow-up) by group.

**Cooking confidence components^**2**^**	**Interaction effect**		**Control Group (*****n*** **=55)**				**Intervention Group (*****n*** **=** **176)**			
	***p-*value^**3**^**	**Effect size**^**4**^ **($\eta_{p}^{2}$)**	**CT1** **mean (SE)**	**CT2** **mean (SE)**	**Change (CT2-CT1)** **mean (SE)**	***Post-hoc*** ***p value***^**3**^	**IT1** **mean (SE)**	**IT2** **mean (SE)**	**Change (T2-T1)** **mean (SE)**	***Post-hoc*** ***p*-value**^**3**^
Confidence about being able to cook from basic ingredients	<0.001^**^	large effect (0.12)	3.61 (0.16)	3.66 (0.12)	0.05 (0.13)	0.652	3.54 (0.10)	4.36 (0.08)	0.82 (0.08)	<0.001^**^
Confidence about following a simple recipe	0.004^**^	Small-medium effect (0.04)	4.00 (0.13)	4.23 (0.10)	0.23 (0.11)	0.036^*^	4.03 (0.08)	4.61 (0.06)	0.57 (0.07)	<0.001^**^
Confidence about preparing and cooking new foods and recipes	<0.001^**^	medium effect (0.07)	3.43 (0.16)	3.69 (0.16)	0.26 (0.14)	0.070	3.40 (0.10)	4.29 (0.08)	0.89 (0.09)	<0.001^**^
Confidence that was is cooked will “turn out” well	<0.001^**^	medium effect (0.07)	3.21 (0.15)	3.39 (0.19)	0.18 (0.13)	0.147	3.27 (0.09)	3.98 (0.07)	0.72 (0.08)	<0.001^**^
Confidence about tasting foods not eaten before	0.004^**^	Small-medium effect (0.04)	3.77 (0.16)	3.90 (0.13)	0.13 (0.12)	0.291	3.58 (0.10)	4.10 (0.08)	0.52 (0.08)	<0.001^**^
Overall confidence score^5^	<0.001^**^	large effect (0.11)	18.01 (0.62)	18.87 (0.48)	0.86 (0.50)	0.069	17.82 (0.38)	21.34 (0.30)	3.52 (0.29)	<0.001^**^

*Values reported are adjusted for the effects of age, gender, BMI, income and education*.

2 *Responses provided on a 5-point Likert scale ranging from 1 = “Not confident at all” to 5 = “Extremely confident”*.

3* *p < 0.05*

** *p < 0.01*.

4 *Partial eta squared effect size ranges: small effect = 0.00–0.01; medium effect = 0.01–0.06; large effect = 0.06–0.14*.

5 *Sum of five confidence question responses provided on a 5-point Likert scale ranging from 1 = “Not confident at all” to 5 = “Extremely confident”*.

### Dietary Behavior

Results for the secondary outcome measures examined between the control and intervention groups over time are displayed in Table 3 and Supplementary Material Additional File 5.

**Table 3:** Multivariable-adjusted general linear models of program interaction effects over time on secondary outcome measures at T1 (baseline) and T2 (5/7-week follow-up) by group.

**Secondary outcome measure**	**Interaction effect**		**Control Group (*****n*** **=55)**				**Intervention Group (*****n*** **=** **176)**			
	***p* value^1^**	**Effect size**^2^ **($\eta_{p}^{2}$)**	**CT1** **mean (SE)**	**CT2** **mean (SE)**	**Change (CT2-CT1)** **mean (SE)**	***Post-hoc*** ***p*-value**	**IT1** **mean (SE)**	**IT2** **mean (SE)**	**Change (T2-T1)** **mean (SE)**	***Post-hoc*** ***p*-value**
Healthy cooking^3^ In a typical week, how often do you prepare and cook a *main meal* from basic ingredients?	0.012^*^	Small-medium effect (0.03)	4.21 (0.17)	4.29 (0.15)	0.05 (0.12)	0.704	4.18 (0.10)	4.56 (0.09)	0.38 (0.08)	<0.001^**^
Attitudes and beliefs regarding healthy eating^4^ **a)** I find it easy to change my eating habits	0.010^*^	Small-medium effect (0.03)	2.52 (0.12)	2.53 (0.10)	0.01 (0.10)	0.883	2.52 (0.07)	2.80 (0.06)	0.28 (0.06)	<0.001^**^
**b)** My lifestyle prevents me eating a healthy diet	0.047^*^	Small effect (0.02)	3.10 (0.11)	3.05 (0.10)	−0.04 (0.12)	0.703	3.24 (0.07)	3.44. (0.06)	0.20 (0.07)	0.005^**^
**e)** I can create a healthy meal from scratch in 30 min	0.017^*^	Small-medium effect (0.03)	2.81 (0.13)	3.03 (0.11)	0.22 (0.12)	0.063	2.77 (0.08)	3.29 (0.07)	0.53 (0.07)	<0.001^**^
**f)** I enjoy cooking	<0.001^**^	Medium effect (0.06)	3.22 (0.14)	3.10 (0.12)	−0.12 (0.09)	0.152	3.08 (0.09)	3.30 (0.07)	0.22 (0.05)	<0.001^**^
**g)** I enjoy cooking for others	<0.001^**^	Medium effect (0.05)	3.13 (0.14)	3.05 (0.12)	−0.08 (0.09)	0.395	3.00 (0.08)	3.27 (0.07)	0.28 (0.06)	<0.001^**^
**h)** I get a lot of satisfaction from cooking my meals	0.007^**^	Small-medium effect (0.03)	3.05 (0.14)	3.10 (0.11)	0.052 (0.10)	0.584	2.97 (0.08)	3.30 (0.07)	0.33 (0.06)	<0.001^**^
GSE^5^	Not significant		31.9 (0.85)	32.3 (0.79)	0.38 (0.55)	0.492	32.4 (0.52)	33.5 (0.48)	1.11 (0.34)	0.001^*^
General health^6^ In general, how do you feel about your health?	0.022^*^	Small effect (0.02)	3.06 (0.12)	3.10 (0.13)	0.04 (0.12)	0.726	3.10 (0.08)	2.84 (0.08)	−0.26 (0.08)	<0.001^**^
SF-12 MCS score^7^	0.033^*^	small effect (0.02)	49.7 (1.4)	48.2 (1.4)	−1.53 (1.08)	0.158	49.6 (0.88)	50.6 (0.88)	0.94 (0.67)	0.158
Subjective vitality scale total	0.034^*^	small effect (0.02)	26.3 (1.2)	26.7 (1.1)	0.43 (0.73)	0.560	26.2 (0.71)	28.3 (0.70)	2.09 (0.45)	<0.001^**^
WEMWBS^8^	Not significant		50.49 (1.24)	50.47 (1.30)	−0.02 (0.92)	0.981	50.50 (0.76)	52.08 (0.80)	1.58 (0.56)	0.005^**^

*Values reported are adjusted for the effects of age, gender, BMI, income and education*.

1* *p < 0.05*

** *p < 0.01*.

2 *Partial eta squared effect size ranges: small effect = 0.00–0.01; medium effect = 0.01–0.06; large effect = 0.06–0.14*.

3 *Categorical response items where 1 = “Never”; 2 = ‘Less than once'; 3 ='Once'; 4 ='2-3 times; 5 ='4-6 times'; 6 ='daily'*.

4 *Likert scale response where 1='strongly disagree'; 2 ='somewhat disagree'; 3 ='somewhat agree'; 4 ='strongly agree'*.

5 *Rosenburg's Global Self Esteem score, Likert scale response where 1='strongly disagree'; 2 ='somewhat disagree'; 3 ='somewhat agree'; 4 ='strongly agree'*.

6 *Categorical response items where 1 = ‘Excellent'; 2 = ‘Very good'; 3 ='Good'; 4 ='Fair'; 5 ='Poor'*.

7 *SF-12 Australian norm-based Mental Component Summary score*.

8 *Warwick Edinburgh Mental Wellbeing Scale total score*.

#### Cooking Enjoyment, Satisfaction and Ability

Participants in the intervention group reported an increase in their enjoyment of cooking generally and cooking for others post-program that was sustained over 6 months and was not observed for the control group (Table 3; Supplementary Material Additional File 4). There were similar results for cooking satisfaction, frequency of cooking a main meal from basic ingredients and the ability to do so in 30 min. For all three outcomes, there were significant improvements reported by those in the intervention group that were not found in the control group and all were maintained at 6-month follow-up (Table 3; Supplementary Material Additional File 4). Participants' responses to whether they thought their lifestyle prevented their ability to eat healthily and whether they found it easy to change their eating habits also saw positive gains for the intervention group post-program. For both outcomes, the improvement was sustained after 6 months and was not observed for participants in the control group (Table 3; Supplementary Material Additional File 4).

#### Healthy Eating and Food Expenditure

No significant differences were observed between the intervention and control group across the three measures of healthy eating; intake of vegetables, intake of fruit, and consumption of take away/fast foods (Supplementary Material Additional File 5). On average, both groups reported consuming 2–3 serves of vegetables per day and 1–2 serves of fruit per day and this did not change from T1 to T2. Participants from both groups, consumed takeaway food 4–6 times a month on average at each time-point they were surveyed (Supplementary Material Additional File 5). No significant change in household spending habits was observed. At the outset, both groups spent ~$100–200 per week on total food and drinks per household. Across the groups, the total household spend on fruit and vegetables, on average, was between $20 and $50 per week and between $10 and $40 per week on take away/fast foods. No significant changes were observed at 6-month follow-up (Supplementary Material Additional File 5).

#### Social Connectedness

For the three measures of social connectedness around cooking and eating, responses for both groups at T1 and T2 were not significantly different. Overall, participants ate their evening meal with others in their household ~2–4 times per week. Across both groups, dinner was eaten in front of the TV on average twice per week and sitting at a dinner table on average 2–3 times per week. No significant changes were observed at 6-month follow-up (Supplementary Material Additional File 5).

#### Attitudes and Beliefs

The program did not significantly affect participants' attitudes and beliefs surrounding acquisition and consumption of fruit and vegetables. Overall, participants in both groups reported a moderate agreement about eating enough fruit and vegetables; had a moderate to strong agreement that vegetables can be made tasty; and that fruit and vegetables are cheaper in season. No significant changes in these components were observed for either group at 6-month follow-up (Supplementary Material Additional File 5). Similar results were observed for responses regarding the affordability of a healthy meal, including meal preparation from low-cost ingredients and affordability of purchasing more fruit and vegetables. Pre- and post-program, both groups somewhat agreed with the statements regarding beliefs about meal preparation from basic low-cost ingredients and economic constraints to buying more fruit and vegetables (Supplementary Material Additional File 5).

#### General and Mental Health and Wellbeing

In the intervention group, participants' self-reported general health, GSE, mental health (WEBMWS) and subjective vitality (SVS) all significantly improved post-program (Table 3), whilst no significant changes for any of the tests were observed in the control group. At baseline, 32% of participants in the control group and 34% in the intervention group had MCS scores below the Australian population average (47). At T2 for the intervention group there were 8% fewer participants with <47 MCS scores which was a significant improvement (Table 3). The control group experienced a 2% decrease in those with below population average MCS score at T2 and therefore the between group difference was not significant (Table 3). For all mental health and wellbeing measures there were no significant changes observed at T3, therefore all improvements were sustained over 6 months (Supplementary Material Additional File 4).

#### Nutrition Knowledge

Unadjusted results for the program's effect on self-reported nutrition knowledge demonstrated no significant difference within groups, or over time. At T1, combined averages for correct answers to the three questions regarding salt, fat and sugar were 80% for all; 83% for the control group; and 78% for the intervention group. At T2, they were 82% for all; 80% for the control group; and 82% for the intervention group (Supplementary Material Additional File 2).

## Discussion

This study found that all five components of cooking confidence increased for participants in the intervention group compared to the control group. Post-program (T2) the participants reported increased confidence in preparing and cooking a main meal from basic ingredients; following a recipe; and tasting new foods, that were sustained over the ensuing 6 months. Previous programs in other Australian jurisdictions that have implemented the JMOF program, in either a fixed or mobile kitchen mode, had similar findings (62, 74).

It has been suggested that where program implementation includes learning through observation, interaction and reflection, confidence is more likely to be fostered (50, 84, 85). Furthermore, it is understood that to achieve the best outcomes for improving knowledge and initiating behavior change, the individual should actively participate in the experience. During which, they should be provided with the skills to undertake the experience, have the opportunity to innovate and should reflect on it afterwards (54, 84). The JMOF program's positive effect on cooking confidence may be due to the program's scaffolded design and deliberate practical application of cooking skills to daily lives (86) and may also have provided the impetus to engage with the program initially. Moreover, the program's experiential learning model (84) resulted in positive outcomes for the ability to overcome lifestyle barriers to healthy eating such as lack of confidence and satisfaction around cooking. Evidence suggests that increased self-efficacy about being able to create a healthy meal from scratch may have a positive influence over dietary habits about cooking meals from basic ingredients (8, 13). These findings highlight the value of building cooking confidence to enable the use of basic ingredients, which could foster healthier eating habits and reduce the need to rely on highly processed foods in home cooking (10, 54, 87). Ideally, to support this premise, we should have observed improvements in healthy eating. However, the study was limited by the basic data collected regarding habits surrounding fruit and vegetable acquisition and consumption and frequency of fast-food/takeaway meals (4 questions covering fruit, vegetables and fast-foods/take-away), which for this study determined healthy eating. We did not detect a significant change in these habits post-program. As change to dietary behavior is an important outcome for food literacy cooking programs (8), future analysis of the broader ECU study will investigate the effects of the program on dietary intake in greater detail in conjunction with dietary and gut biomarkers (68). It is now well-known that diet is a major factor in gut microbiome balance and can considerably influence ensuing interactions with host health (19, 88). Western diets, low in FV and DF and high in discretionary foods, can have a detrimental effect on gut microbiome composition that is associated with poorer mental and physical health (31, 89–92). The broader study will explore the relationship between cooking confidence, diet quality and the gut microbiome by looking at diet and gut biomarkers that are implicated in mental health via healthy signaling through the gut-brain axis.

Some research has found there is a correlation between education status and food literacy, such that those with higher education are more likely to be food literate, have a greater access to food and a higher quality of diet (65, 93). However, Wolfson et al. reported that higher education, higher income and urban living were the leading factors associated with less frequent home cooking (94). We found no significant differences between participants with high school or lower education (36%) and those with some form of tertiary education, for gains in cooking ability and cooking confidence. Similar results have been reported in other cooking program studies (48). Worsley et al. (95) found that socio-demographic factors such as education did not predict who wanted to learn to cook and that social influences were more significant. The commonality of a desire to be able to create a healthy meal from inexpensive basic ingredients in a short space of time was the main driver (95). This raises speculation over whether education is the strong predictor of food literacy as once thought, or that the opportunity to participate in learning experiences has a more profound influence (96, 97). In Australia, the education curriculum includes cooking and nutrition education in primary and secondary schools as part of the Health and Physical Learning (HPE) area (98). However, although valued as highly as subjects in health, physical education and digital technology, by students and parents alike, this is limited and variable across schools (99, 100). It is a mandated learning area, yet the delivery of these curriculum-based activities is linked to teacher expertise and confidence with teaching cooking and nutrition (99, 100). Regardless, the literature states that higher food literacy is an important predictor for healthy dietary intake (101), and in a longitudinal study, Dutch researchers found those with adequate cooking skills in emerging adulthood had greater likelihood of frequent fruit and vegetable intake and fewer barriers to healthy food preparation 10 years later (102).

Nearly two-thirds of the participants in this study were overweight or obese and an obesogenic environment has been linked to dieting, gender and self-esteem that is particularly enhanced during adolescence (103–106). Although we found no difference in confidence gains between participants who were a healthy weight and those who were overweight or obese, there were significant improvements for self-reported mental health and wellbeing that were observed in the intervention group post-program but not in the control group. The link between mental health and obesity has been well-studied (107, 108) with findings showing that those with better mental health have greater self-efficacy to manage their weight and adopt a healthier lifestyle (109). There is also evidence that social connectedness is supportive of public mental health (110), whereas lower food literacy and unhealthy dietary patterns are associated with poor mental health and wellbeing (20). Although no change was observed for habitual weekly consumption of fruit and vegetables; take-away meals; or to social connectedness surrounding mealtimes, the positive outcomes for mental health could be attributed to the improvements in cooking confidence, satisfaction and self-efficacy surrounding plan, manage and select domains of food literacy. These improvements may also be due act of participating in a group activity which has been shown to benefit psychosocial outcomes (111). In both groups, two-thirds of participants attended the program as part of a group or with others and this may explain why there were no significant changes to questions surrounding social-connectedness. A recent Canadian study exploring food literacy amongst youths involved in a food literacy school kitchen garden program, found participation in the program resulted in positive outcomes for mental health and wellbeing (97). The youths acknowledged improvements in their social health due to engagement and activity with others (97). The experience of growing the food and being in nature led to improvements in their physical health and a greater interest in eating healthily (97). In consideration of COVID-19, social distancing, on-line delivery over face-to-face learning platforms and the related rise in mental health conditions, the need to focus on social connectedness is ever more important (111).

In a further exploration of differences between genders, our study found that male participants experienced greater gains in cooking confidence than their female counterparts. Most households possess a food “gatekeeper” who is the primary person responsible for planning, purchasing and cooking meals for the household (57). The confidence of the gatekeeper plays a critical role in how healthy the food environment is within the home (57, 112). The traditional role of women as gatekeepers has shifted in recent years, due partly to an increase in women entering the workforce and also to changes in the economy (112, 113). However, a recent global study comparing frequency of cooking at home between genders, found that cooking at home is still a highly gendered task (94). Australian females cooked an evening meal 4–5 times per week where males cooked only 2–3 times (94) which the literature suggests may be associated with a lack of cooking confidence (57). In our study at the commencement of the program, the female participants (*n* = 77%) were more confident about cooking than males (*n* = 23%). By the end of the program, however, both genders had equal confidence about their cooking skills and recipe use. The experiential learning model underpinning this program's design plus the convivial group setting, may have contributed to changes in attitude toward the kitchen environment that was more emphasized in those with lower pre-program confidence (55). These changes could support change to the household food environment by reducing the gender bias and leading to more frequent home cooking by males in the home (10, 94, 114). This in turn may help to overcome the barriers presented by lack of “knowing how to cook” and the imposing influence of time constraints (56, 57).

### Limitations

Defining cooking confidence is not straightforward and measuring self-reported cooking confidence can be contentious as it could be interpreted as the ability to follow a recipe; measure and prepare ingredients; or as the ability to use a variety of different cooking methods (8). Furthermore, the use of self-report measures, such as those included in the LAB survey, is subject to bias such as social desirability bias (50, 115). Similarly, our study may have been limited by sampling bias due to the over-representation of those with pre-existing higher than average cooking confidence, greater interest in food and nutrition and motivation toward healthy cooking and eating. Thus, the sensitivity of the tools to demonstrate the program efficacy may be impacted by a ceiling effect (116). Wallace et al. (117) suggest that those who enroll in cooking programs, do so because they recognize the importance of nutrition as a modifiable factor in the maintenance of health and essential for the prevention of chronic disease. This type of bias has been raised as a criticism of food-literacy cooking interventions (53, 117, 118). In our study, social-desirability bias may also have contributed to the above-average responses for questions surrounding healthy eating; healthy cooking; weekly food shopping spending habits; social-connectedness around food; and nutrition knowledge. This could explain the lack of intervention effect and no between-group differences. Due to the online nature of the LAB survey, the attrition rate at T3 was relatively high (16%) so that the final sample of complete cases may be biased and therefore generalisability of the findings is cautioned. However, the 6-month results for sustainability of program effect were similar to those for earlier evaluations even though in these studies the follow-up rates were higher [31% in Ipswich (62) and 27% in Victoria (73)]. In our study, the ratio of control to intervention participants and the sample size were both lower than reported previously. In addition, due to the program logistics, it was only feasible to run a 5-week control period and a 7-week intervention period that was different to those for earlier studies and may present a limitation. The timeline for each class and the duration of the mobile kitchen's visit to each location was determined by TGF and therefore beyond our control. In some instances, there were delays in course promotion that meant a shorter lead-up time, which reduced the number of registrants available for both control and intervention group recruitment. Implementation of programs such as these are demanding; logistically, economically and physically; yet the broad-reaching, potential benefits of increasing cooking skills and cooking confidence within the community are evident (119). The typical demographic characteristics of the participants may have been skewed by the fact that the mobile kitchen was situated at the same university campus 6 times out of the 16 and in this location, recruitment may have reached saturation point.

Final limitations may have been due to the waitlist control. Concerns have been raised that this type of study design can lead to over-estimated intervention effects (120). Even though the control participants in our study, did not go on to become intervention participants due to time restraints, the situations where outcomes were experienced by both the control and the intervention group may be explained by the Stages of Change theory (121). Control group participants selected from the waitlist had committed to the program and in doing so, instigated behavior change and moved from pre-contemplation to change/action stage by the act of registering and had, therefore, already shifted behavior (117). Similar findings were reported in the previous Ipswich evaluation where a positive change within both groups was also observed (74).

### Strengths

The significant improvements that were observed in perceived general health, mental health and subjective vitality as a result of participation are the important findings from this study. They suggest a tentative link between cooking confidence and satisfaction around cooking, and mental health benefits. We also found that as a result of participation in this scaffolded, experiential program, all participants experienced significant gains in cooking confidence and through sensitivity analyses we found these gains were greater for males than females. Further, the positive outcomes observed for the intervention group were maintained for 6 months post program. Cooking satisfaction, enjoyment and ability, that have been associated with healthier eating behaviors, were all improved for intervention participants in our study. The lack of intervention effect for fruit and vegetable consumption, nutrition knowledge, healthy eating and expenditure, highlight the importance of targeting future cooking education sessions at population groups that will experience the greatest gains. Begley et al. (53) express the importance of looking beyond dietary behavior change when investigating the benefits of a food literacy cooking program such as this one. The mixed results of this investigation contribute to the growing evidence required to create effective programs in the future (53).

## Conclusions

The positive findings surrounding mental health, subjective vitality and general health observed in this study are encouraging. Whether these improvements were due to improved cooking confidence or being involved in a group activity cannot be determined here, regardless the findings merit further research into the potential to include food literacy cooking interventions as a preventive measure for population mental health programs. To overcome current issues raised by the global pandemic, this could be achieved via digital translation. This was demonstrated by a recent bi-centre randomized controlled trial where delivery of a culinary coaching telemedicine program was found to be an effective intervention for teaching home cooking skills that improved participant wellbeing (122).

The case for targeting improved diet quality in the population as a preventive strategy to halt or slow the rise in poor mental health, obesity and other metabolic health disorders has been reinforced by the findings (35, 123). However, implementation research is needed to ensure that outcomes are a true reflection of an individual's food environment. Future programs should continue to prioritize the barriers to healthy eating such as time restrictions, and place greater emphasis on the value of healthy eating/avoidance of ultra-processed convenience foods over quick and easy home-cooked meals (96, 119, 124). To increase the long-term benefits of these programs, when planning interventions, it is recommended that elements from a sustainability framework should be included, similar to those recommended by Whelan et al. (125). In addition, to help promote translation of skills into everyday behaviors, skills interventions that have proved to be successful have included either a weekly session with a dietitian or qualified nutritionist (64, 119), or an information session on the gut microbiome and the importance of gut health (126). These education sessions should provide an explanation of *why* fresh whole foods are better than processed convenience foods, for both physical and mental health (49, 99). Moreover, if these topics are introduced during schooling, it may help to overcome some of the barriers faced in later life and provide a sound knowledge base from which to navigate the quantities of misinformation that flood social media. Other programs that have led to success include participation in community garden schemes with an additional component of cooking one's own produce (97). Finally, to support effective translation and uptake of the more relevant findings and ensure greater impact in the community, research should be linked to key governmental directives.

## Data Availability Statement

The raw data supporting the conclusions of this article will be made available by the authors, without undue reservation.

## Ethics Statement

The studies involving human participants were reviewed and approved by Edith Cowan University Human Research Ethics Committee. The patients/participants provided their written informed consent to participate in this study.

## Author Contributions

AD, RS, CC, MB, and RN contributed to the conception and design of the study. JR, SB, and AD were responsible for implementation. SF and JLo provided statistical expertise. JR, SF, JLo, RS, JLe, CC, MB, SB, and AD contributed to refinement of the evaluation and the writing and editing of the manuscript. All authors have read and approved the manuscript.

## Funding

This research received no external funding from any agency in the public, commercial, or not-for-profit sectors. The study was self-supported by ECU, as the Major Partner – Western Australia, by providing funding to The Good Foundation for the provision of a Jamie's Ministry of Food Australia Mobile Kitchen at ECU campuses and other agreed Western Australian locations across a 3-year period. ECU also funded all utility costs associated with the program when held at ECU campuses, and the research study in its entirety. The salary of JLe is supported by a National Heart Foundation of Australia future leader fellowship (102817).

## Conflict of Interest

The authors declare that the research was conducted in the absence of any commercial or financial relationships that could be construed as a potential conflict of interest.

## Publisher's Note

All claims expressed in this article are solely those of the authors and do not necessarily represent those of their affiliated organizations, or those of the publisher, the editors and the reviewers. Any product that may be evaluated in this article, or claim that may be made by its manufacturer, is not guaranteed or endorsed by the publisher.

## Abbreviations

SF-12 MCS: SF-12 health survey mental component summary score
WEMWBS: warwick edinburgh mental wellbeing scale
SVS: Subjective vitality scale
NHS: national health survey
JMOF: jamie's ministry of food
WA: western Australia
TGF: the good foundation
ECU: edith cowan university
ECU SRC: ECU survey research Centre
HREC: human research ethics committee
RGSE: rosenburg global self esteem scale
GLM: general linear modeling
T1, T2, T3: timepoints 1, 2 and 3.
