# Supplementary material for: How a 7-Week Food Literacy Cooking Program Affects Cooking Confidence and Mental Health: Findings of a Quasi-Experimental Controlled Intervention Trial
Source: Front Nutr. 2022 Mar 17;9:802940. doi: 10.3389/fnut.2022.802940 (PMC8970183; doi:10.3389/fnut.2022.802940)
Supplement: Supplementary file 1 [file Data_Sheet_1.pdf]

# *Supplementary Material*

**Supplementary material - Additional File 1. Part a.** Study timetable of program dates and course locations throughout study period

| Site | Site Type (visit number) | Course Dates | Course Release Date | Control Date | Intervention Date |
|---|---|---|---|---|---|
| 1 | Metro site A (1) | 12/09/16-29/10/16 | 04/07/2016 | 08/08/2016 | 12/09/2016 |
| 2 | Metro site B (1) | 03/11/16-21/12/16 | 25/08/2016 | 29/09/2016 | 03/11/2016 |
| 3 | Regional site A (1) | 16/1/17-18/02/17 | 12/12/2016 | N/A* | 16/01/2017 |
| 4 | Metro site A (2) | 22/02/17-11/04/17 | 26/12/2016 | 18/01/2017 | 22/02/2017 |
| 5 | Metro site A (3) | 19/04/17-06/06/17 | 22/02/2017 | 15/03/2017 | 19/04/2017 |
| 6 | Metro site C (1) | 12/06/17-29/07/17 | 10/04/2017 | 08/05/2017 | 12/06/2017 |
| 7 | Regional site B (1) | 07/08/17-23/09/17 | 12/06/2017 | 03/07/2017 | 07/08/2017 |
| 8 | Metro site D (1) | 27/09/17-14/11/17 | 02/08/2017 | 23/08/2017 | 27/09/2017 |
| 9 | Metro site E (1) | 20/11/17-23/12/17 | 25/09/2017 | 16/10/2017 | 20/11/2017 |
| 10 | Regional site A (2) | 08/01/18-25/2/18 | 13/11/2017 | 04/12/2017 | 08/01/2018 |
| 11 | Metro site A (4) | 02/03/18-24/04/18 | 05/01/2018 | 26/01/2018 | 02/03/2018 |
| 12 | Metro site A (5) | 26/04/18-13/6/18 | 01/03/2018 | 22/03/2018 | 26/04/2018 |
| 13 | Metro site F (1) | 18/06/18-21/07/18 | 16/04/2018 | 14/05/2018 | 18/06/2018 |
| 14 | Regional site C (1) | 30/70/18-31/08/18 | 04/06/2018 | 25/06/2018 | 30/07/2018 |
| 15 | Regional site B (2) | 05/09/18-23/10/18 | 11/07/2018 | 01/08/2018 | 05/09/2018 |
| 16 | Metro site A (6) | 01/11/18-18/12/18 | 30/08/2018 | 27/09/2018 | 01/11/2018 |

*No Control due to Christmas

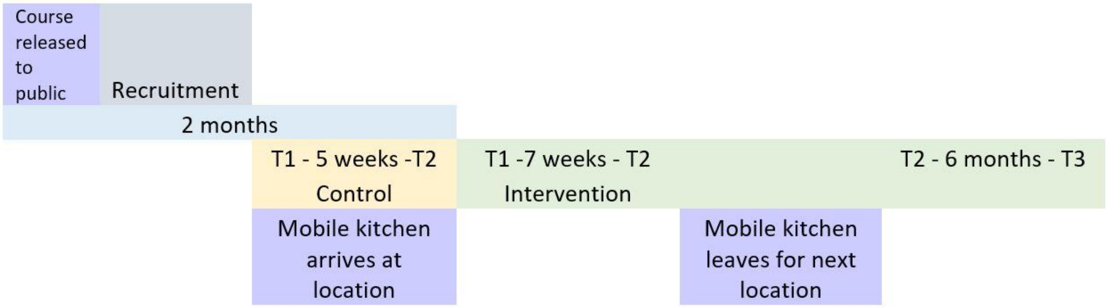


## Part b. Timeline for each location

**Supplementary material – Additional File 2.** LAB survey questions surrounding secondary outcome measures

HEALTHY COOKING:

[Never; Less than once; Once; 2-3 times; 4-6 times; Daily]

- In a typical week, how often do you prepare and cook a main meal from basic ingredients?
- In a typical week, how often do you eat ready-made meals at home?
- In a typical week, how often do you include salad or vegetables with the main meal?

HEALTHY EATING:

[Never; Less than once; Once; 2-3 times; 4-6 times; Daily]

- How often do you include salad or vegetables with the main meal? For example, chicken with vegetables or pasta with salad.
- How many serves of vegetables including fresh, dried, frozen and tinned vegetables do you usually eat each day, where a serve is half a cup of cooked vegetables or 1 cup of salad?
- How many serves of fruit including fresh, dried, frozen and tinned vegetables do you usually eat each day, where a serve is a medium piece or two small pieces of fruit, or a cup of diced pieces?
- How many times do you eat take-away or 'fast-foods' from places such as McDonalds, Red-Rooster, fish and chip shop, Asian take-away or local take-away pizza places?

AFFORDABILITY OF A HEALTHY MEAL

- In a typical week, how much does your household spend in total on food and drink?
- In a typical week, how much does your household spend in total on fruit and vegetables?
- In a typical week, how much does your household spend in total on take away/fast foods?

SOCIAL CONNECTEDNESS AROUND COOKING AND EATING

[Never; Less than once; Once; 2-3 times; 4-6 times; Daily]

- In a typical week, how often do you eat together at home, with the other people in your household in the evenings?
- In a typical week, how often do you eat your dinner in front of the TV?
- In a typical week, how often do you eat your dinner sitting at a dinner table or equivalent?

ATTITUDES AND BELIEFS

- Please choose the answer which best indicates how much you agree or disagree with each of the following statements. [Strongly disagree; somewhat disagree; somewhat agree; strongly agree]
- I find it easy to change my eating habits
- My lifestyle prevents me eating a healthy diet
- Vegetables can be tasty foods
- I eat enough fruit and vegetables
- I can put together a healthy meal from scratch in 30 minutes

- I enjoy cooking
- I enjoy cooking for others
- I get a lot of satisfaction from cooking my meals
- I enjoy eating a meal with others
- I can prepare a meal from basic ingredients that is low in price
- Fruit and vegetables are cheaper when they are in season
- Buying more fruit and vegetables would be difficult on my budget

NUTRITION KNOWLEDGE

- Which of the following choices do you think is not high in salt and could be used to enhance the flavours in food?

[Bacon, Cheese, Tomato sauce, Herbs and spices, Once a week, Not sure]

- Out of the items listed below, which foods do you think health experts recommend eating the least?

[Olive oil, Butter, Avocados, Nuts and seeds, Vegetable oil, Not sure]

- If a person felt like something sweet, but was trying to cut down on sugar, which would be the best choice?

[Glass of fruit juice, A piece of fruit, A plain biscuit, A cereal snack bar, Not sure]

**Supplementary material – Additional File 3.** Comparison of demographic characteristics by group at T1 and comparison of means at T2.

| | **T1 Control** ***n*** **(%)** | **T1 Intervention** ***n*** **(%)** | **T1** $\chi^2$ **Test** ***p*** **value**[1] | **T2** $\chi^2$ **Test** ***p*** **value**[1] |
|---|---|---|---|---|
| Male | 39 (23.8) | 111 (22.5) | 0.738 | |
| Female | 125 (76.2) | 382 (77.5) | | 0.378 |
| #Age | 44.4 ±14.9 | 43.9 ±14.7 | 0.698 | 0.886 |
| #BMI kg/m$^2$ | 27.1 ± 6.2 | 27.5 ± 6.1 | | 0.103 |
| Speaks English as the main language at home | 143 (87.2) | 453 (91.9) | 0.073 | 0.157 |
| Completed education beyond Year 12 high school | 110 (67.0) | 314 (63.7) | 0.556 | 0.552 |
| Occupation | | | | |
| Working full-time | 51 (31.1) | 148 (30) | | |
| Working part-time | 44 (26.8) | 139 (28.2) | | |
| Full-time student | 20 (12.2) | 35 (7.1) | | |
| Part-Time student | 5 (3.0) | 13 (2.6) | | |
| Home duties/carer | 12 (7.3) | 49 (9.9) | | |
| Not working (unemployed, retired, unable to work and others) | 32 (19.5) | 109 (22.1) | | |
| Household yearly income >$50,000 | 96 (67.1) | 290 (68.1) | 0.684 | 0.218 |
| Access to a kitchen to prepare own meals | 163 (99.4) | 492 (99.8) | 0.413 | 0.576 |
| Attending participant does household food shopping | 138 (82.9) | 418 (84.8) | 0.570 | 0.694 |

[1] Significance defined as *$p<0.05$ and **$p<0.01$, #Mean ±SD

**Supplementary material – Additional File 4.** Program interaction effects over time for outcome measures from T2 to T3 (6 months post-program) in intervention group

| **Cooking confidence** | **Change (T3-T2) mean (SE[2])** | **Post Hoc *p* value[3]** |
|---|---|---|
| Confidence about being able to cook from basic ingredients | 0.006 (0.08) | 0.94 |
| Confidence about following a simple recipe | 0.146 (0.09) | 0.10 |
| Confidence about preparing and cooking new foods and recipes | 0.010 (0.10) | 0.92 |
| Confidence that was is cooked will "turn out" well | 0.069 (0.11) | 0.54 |
| Confidence about tasting foods not eaten before | 0.047 (0.12) | 0.69 |
| **Healthy cooking** | | |
| In a typical week, how often do you prepare and cook a main meal from basic ingredients? | 0.005 (0.11) | 0.96 |
| In a typical week, how often do you eat ready-made meals at home? | 0.125 (0.16) | 0.42 |
| In a typical week, how often do you include salad or vegetables with the main meal? | 0.21 (0.13) | 0.10 |
| **Healthy eating** | | |
| How many serves of vegetables, including fresh, dried, frozen and tinned vegetables do you usually eat each day, where a serve is half a cup of cooked vegetables or 1 cup of salad? | 0.08 (0.18) | 0.67 |
| How many serves of fruit, including fresh, dried, frozen and tinned fruit do you usually eat each day, where a serve is a medium piece or two small pieces of fruit, or a cup of diced pieces? | -0.10 (0.16) | 0.534 |
| How many times do you eat take-away or 'fast foods'? | -0.047 (0.09) | 0.597 |
| **Affordability of a healthy meal** | | |
| In a typical week, how much does your household spend in total on food and drink? | -0.19 (0.13) | 0.15 |
| In a typical week, how much does your household spend in total on fruit and vegetables? | -0.12 (0.65) | 0.46 |
| In a typical week, how much does your household spend in total on take away/fast foods? | 0.033 (0.24) | 0.89 |
| **Social connectedness around cooking and eating[4]** | | |
| In a typical week, how often do you eat together at home, with the other people in your household in the evenings? | 0.58 (0.33) | 0.09 |
| In a typical week, how often do you eat your dinner in front of the TV? | -0.002 (0.15) | 0.99 |

| | | |
|---|---|---|
| In a typical week, how often do you eat your dinner sitting at a dinner table or equivalent? | 0.11 (0.18) | 0.56 |
| **Attitudes and beliefs regarding healthy eating[5]** | | |
| I find it easy to change my eating habits | -0.03 (0.11) | 0.794 |
| My lifestyle prevents me eating a healthy diet^ | 0.02 (0.11) | 0.85 |
| Vegetables can be tasty foods | 0.03 (0.07) | 0.71 |
| I eat enough fruit and vegetables | 0.08 (0.10) | 0.43 |
| Fruit and vegetables are cheaper when they are in season | 0.006 (0.09) | 0.95 |
| **Cooking ability, enjoyment and satisfaction[5]** | | |
| I can put together a healthy meal from scratch in 30 minutes | 0.06 (0.09) | 0.56 |
| I enjoy cooking | -0.034 (0.08) | 0.688 |
| I enjoy cooking for others | -0.09 (0.08) | 0.252 |
| I get a lot of satisfaction from cooking my meals | -0.13 (0.10) | 0.214 |
| I enjoy eating a meal with others | 0.03 (0.08) | 0.723 |
| **Attitudes & beliefs of affordability of a healthy meal[5]** | | |
| I can prepare a meal from basic ingredients that is low in price | -0.05 (0.08) | 0.498 |
| Buying more fruit and vegetables would be difficult on my budget^ | -0.10 (0.13) | 0.440 |
| **General health and self-esteem** | | |
| In general how do you feel about your health?[6] | -0.13 (0.11) | 0.228 |
| Global self-esteem | -0.33 (0.53) | 0.531 |
| PCS12 | -0.08 (0.95) | 0.933 |
| MCS12 | -2.37 (1.19) | 0.051 |
| SVS_Total | -1.00 (0.74) | 0.18 |
| WEMWBS | -0.80 (0.87) | 0.360 |

[1]Values reported are adjusted for the effects of age, gender, BMI, income and education
[2]SE = Standard Error of Measurement
[3] **p*<0.05 and ** *p*<0.01
[4]Categorical response item/s where "Never" = 1 "Less than once a month" = 2 "Once a week" = 3 "2 times a week" = 4 "3-4 times a week" = 5 "5 or more times a week" = 6 "Don't know" = Missing = 7
[5]Likert scale response item/s where "Strongly disagree" = 1 "Somewhat disagree" = 2 "Somewhat agree" = 3 "Strongly agree" = 4
[6]Categorical response items where 1 = 'Excellent'; 2 = 'Very good'; 3 = 'Good'; 4 = 'Fair'; 5 = 'Poor'.
^ Item response recoded where "Strongly disagree" = 4 " Somewhat disagree" = 3 "Somewhat agree" = 2 "Strongly agree" = 1

**Supplementary material – Additional File 5.** Multivariable-adjusted general linear models of program interaction effects over time for secondary outcome measures at T1 (baseline), T2 (5/7-week follow-up) and T3 (6-month follow-up – intervention only) by group[1].

| Secondary Outcome Measure | Interaction effect | | Control Group mean (SE[2]) | | | | Intervention Group mean (SE) | | | | | | |
|---|---|---|---|---|---|---|---|---|---|---|---|---|---|
| | *p* value[5] | Effect size[6] (partial eta squared) | Baseline (T1) | Post Program (T2) | Change (T2-T1) | Post Hoc *p* value | Baseline (T1) | Post Program (T2) | Change (T2-T1) | Post Hoc *p* value | Post Program (T3) | Change (T3-T2) | Post Hoc *p* value |
| **Healthy cooking** | | | | | | | | | | | | | |
| In a typical week, how often do you eat ready-made meals at home? | 0.628 | N/S[3] | 2.57 (0.171) | 2.32 (0.17) | 0.25 (0.12) | NA[4] | 2.45 (0.10) | 2.26 (0.10) | -0.18 (0.08) | NA | 2.06 (0.19) | 0.125 (0.16) | 0.42 |
| In a typical week, how often do you include salad or vegetables with the main meal? | 0.127 | N/S | 4.95 (0.14) | 5.16 (0.11) | 0.21 (0.12) | NA[4] | 4.85 (0.08) | 5.24 (0.07) | 0.40 (0.07) | NA | 4.93 (0.11) | 0.21 (0.13) | 0.10 |
| **Healthy eating** | | | | | | | | | | | | | |
| How many serves of vegetables, including fresh, dried, frozen and tinned vegetables do you usually eat each day, where a serve is half a cup of cooked vegetables or 1 cup of salad? | 0.624 | N/S | 4.15 (0.20) | 4.68 (0.20) | 0.53 (0.17) | NA | 4.36 (0.12) | 4.98 (0.12) | 0.62 (0.11) | NA | 4.89 (0.21) | 0.08 (0.18) | 0.67 |
| How many serves of fruit, including fresh, dried, frozen and tinned fruit do you usually eat each day, where a serve is a medium piece or two small pieces of fruit, or a cup of diced pieces? | 0.890 | N/S | 3.54 (0.18) | 3.84 (0.18) | 0.30 (0.15) | NA | 3.49 (0.11) | 3.81 (0.11) | 0.32 (0.09) | NA | 3.69 (0.18) | -0.10 (0.16) | 0.534 |

| **Secondary Outcome Measure** | **Interaction effect** | | **Control Group** mean (SE[2]) | | | | **Intervention Group** mean (SE) | | | | | | |
|---|---|---|---|---|---|---|---|---|---|---|---|---|---|
| | ***p* value[5]** | **Effect size[6] (partial eta squared)** | **Baseline (T1)** | **Post Program (T2)** | **Change (T2-T1)** | **Post Hoc *p* value** | **Baseline (T1)** | **Post Program (T2)** | **Change (T2-T1)** | **Post Hoc *p* value** | **Post Program (T3)** | **Change (T3-T2)** | **Post Hoc *p* value** |
| How many times do you eat take-away or 'fast foods'? | 0.540 | N/S | 2.85 (0.13) | 2.72 (0.11) | -0.12 (0.11) | NA | 2.67 (0.08) | 2.47 (0.07) | -0.20 (0.07) | NA | 2.50 (0.09) | -0.047 (0.09) | 0.597 |
| **Affordability of a healthy meal** | | | | | | | | | | | | | |
| In a typical week, how much does your household spend in total on food and drink | 0.489 | N/S | 5.75 (0.15) | 5.61 (0.15) | -0.14 (0.12) | NA | 5.62 (0.09) | 5.56 (0.10) | -0.05 (0.07) | NA | 5.56 (0.15) | -0.19 (0.13) | 0.15 |
| In a typical week, how much does your household spend in total on fruit and vegetables | 0.814 | N/S | 4.63 (0.16) | 4.79 (0.16) | 0.16 (0.12) | NA | 4.67 (0.10) | 4.80 (0.10) | 0.13 (0.07) | NA | 4.77 (0.16) | -0.12 (0.65) | 0.46 |
| In a typical week, how much does your household spend in total on take away/fast foods | 0.208 | N/S | 3.69 (0.22) | 3.65 (0.21) | -0.05 (0.19) | NA | 3.64 (0.14) | 3.33 (0.13) | -0.31 (0.12) | NA | 3.44 (0.22) | 0.033 (0.24) | 0.89 |
| **Social connectedness around cooking and eating[7]** | | | | | | | | | | | | | |
| In a typical week, how often do you eat together at home, with the other people in your household in the evenings | 0.108 | N/S | 4.53 (0.26) | 4.50 (0.24) | -0.035 (0.16) | NA | 4.74 (0.16) | 4.97 (0.15) | 0.24 (0.10) | NA | 4.89 (0.24) | 0.58 (0.33) | 0.09 |
| In a typical week, how often do you eat your dinner in front of the TV | 0.784 | N/S | 4.29 (0.27) | 4.07 (0.28) | -0.21 (0.15) | NA | 4.04 (0.17) | 3.78 (0.17) | -0.26 (0.09) | NA | 3.82 (0.31) | -0.002 (0.15) | 0.99 |

| Secondary Outcome Measure | Interaction effect | | Control Group mean (SE[2]) | | | | Intervention Group mean (SE) | | | | | | |
|---|---|---|---|---|---|---|---|---|---|---|---|---|---|
| | *p* value[5] | Effect size[6] (partial eta squared) | Baseline (T1) | Post Program (T2) | Change (T2-T1) | Post Hoc *p* value | Baseline (T1) | Post Program (T2) | Change (T2-T1) | Post Hoc *p* value | Post Program (T3) | Change (T3-T2) | Post Hoc *p* value |
| In a typical week, how often do you eat your dinner sitting at a dinner table or equivalent | 0.192 | N/S | 4.22 (0.27) | 4.32 (0.25) | 0.10 (0.14) | NA | 4.20 (0.16) | 4.50 (0.15) | 0.30 (0.09) | NA | 4.28 (0.27) | 0.11 (0.18) | 0.56 |
| **Attitudes and beliefs regarding healthy eating[8]** | | | | | | | | | | | | | |
| Vegetables can be tasty foods | 0.094 | N/S | 3.63 (0.10) | 3.69 (0.08) | 0.06 (0.08) | NA | 3.57 (0.06) | 3.79 (0.05) | 0.21 (0.05) | NA | 3.73 (0.09) | 0.03 (0.07) | 0.71 |
| I eat enough fruit and vegetables | 0.081 | N/S | 2.76 (0.13) | 2.87 (0.11) | 0.11 (0.10) | NA | 2.61 (0.08) | 2.91 (0.07) | 0.30 (0.06) | NA | 2.78 (0.12) | 0.08 (0.10) | 0.43 |
| Fruit and vegetables are cheaper when they are in season | 0.644 | N/S | 3.43 (0.09) | 3.60 (0.08) | 0.17 (0.08) | NA | 3.49 (0.06) | 3.70 (0.05) | 0.21 (0.05) | NA | 3.66 (0.08) | 0.006 (0.09) | 0.95 |
| **Cooking ability[8], enjoyment and satisfaction** | | | | | | | | | | | | | |
| I enjoy eating a meal with others | 0.217 | N/S | 3.54 (0.09) | 3.54 (0.08) | 0.004 (0.09) | NA | 3.46 (0.05) | 3.58 (0.05) | 0.12 (0.05) | NA | 3.55 (0.09) | 0.03 (0.08) | 0.723 |
| **Attitudes & beliefs of affordability of a healthy meal[8]** | | | | | | | | | | | | | |
| I can prepare a meal from basic ingredients that is low in price | 0.161 | N/S | 2.72 (0.12) | 3.05 (0.09) | 0.328 (0.10) | NA | 2.92 (0.07) | 3.40 (0.06) | 0.484 (0.06) | NA | 3.39 (0.09) | -0.05 (0.08) | 0.498 |
| Buying more fruit and vegetables would be difficult on my budget^ | 0.588 | N/S | 3.05 (0.11) | 3.16 (0.10) | 0.11 (0.11) | NA | 3.18 (0.07) | 3.36 (0.06) | 0.067 (0.01) | NA | 3.35 (0.11) | -0.10 (0.13) | 0.440 |

[1]Values reported are adjusted for the effects of age, gender, BMI, income and education; [2]SE = Standard Error of Measurement; [3]N/S = No significant overall interaction effect; [4]Not Applicable as the overall interaction effect is not significant; [5]*$p$<0.05 and ** $p$<0.01; [6]Partial eta squared effect size ranges: small effect = 0.00-0.01; medium effect = 0.01-0.06; large effect = 0.06 – 0.14; [7]Categorical response items where "Never" = 1 "Less than once a month" = 2 "Once a week" = 3 "2 times a week" = 4 "3-4 times a week" = 5 "5 or more times a week" = 6 "Don't know" = Missing = 7; [8]Likert scale response items where "Strongly disagree" = 1 "Somewhat disagree" = 2 "Somewhat agree" = 3 "Strongly agree" = 4; ^ Item response recoded where "Strongly disagree" = 4 " Somewhat disagree" = 3 "Somewhat agree" = 2 "Strongly agree" = 1
